# Sulfasalazine Attenuates Staphylococcal Enterotoxin B-Induced Immune Responses

**DOI:** 10.3390/toxins7020553

**Published:** 2015-02-13

**Authors:** Teresa Krakauer

**Affiliations:** Department of Immunology, Molecular Translational Sciences Division, United States Army Medical Research Institute of Infectious Diseases, Fort Detrick, Frederick, MD 21702-5011, USA; E-Mail: Teresa.krakauer@us.army.mil; Tel.: +1-301-619-4733

**Keywords:** staphylococcal enterotoxin B, inflammatory cytokines, sulfasalazine

## Abstract

Staphylococcal enterotoxin B (SEB) and related exotoxins are important virulence factors produced by *Staphylococcus aureus* as they cause human diseases such as food poisoning and toxic shock. These toxins bind directly to cells of the immune system resulting in hyperactivation of both T lymphocytes and monocytes/macrophages. The excessive release of proinflammatory cytokines from these cells mediates the toxic effects of SEB. This study examined the inhibitory activities of an anti-inflammatory drug, sulfasalazine, on SEB-stimulated human peripheral blood mononuclear cells (PBMC). Sulfasalazine dose-dependently inhibited tumor necrosis factor α, interleukin 1 (IL-1) β, IL-2, IL-6, interferon γ (IFNγ), and various chemotactic cytokines from SEB-stimulated human PBMC. Sulfasalazine also potently blocked SEB-induced T cell proliferation and NFκB activation. These results suggest that sulfasalazine might be useful in mitigating the toxic effects of SEB by blocking SEB-induced host inflammatory cascade and signaling pathways.

## 1. Introduction

Staphylococcal enterotoxin B (SEB) and structurally related bacterial exotoxins are etiological agents that cause a variety of diseases in humans, ranging from food poisoning, autoimmune diseases, and toxic shock [1,2,3]. These exotoxins potently stimulate host immune responses by binding directly to the major histocompatibility complex (MHC) class II molecules on antigen‑presenting cells and specific Vβ regions of the T-cell receptors [4,5]. The staphylococcal exotoxins are also known as superantigens because of their ability to polyclonally activate T cells at picomolar concentrations [1,5]. Their interactions with cells of the immune system result in a massive release of proinflammatory cytokines and chemokines [6,7,8,9,10,11]. These proinflammatory mediators enhance leukocyte migration, promote tissue injury, and coagulation [12,13]. The cytokines, interleukin 1 (IL-1), tumor necrosis factor α (TNFα), and interferon gamma (IFNγ) are pivotal mediators in animal models of superantigen-induced toxic shock [7,11].

Currently, there are no specific drugs available for treating superantigen-induced shock. However, intravenous immunoglobulin is protective if it is administered soon after SEB intoxication [14]. Targeting superantigen-induced host responses with anti-inflammatory drugs is an attractive strategy as some of these compounds block key signaling pathways induced by superantigens and the cytokines induced [15]. Sulfasalazine (SFZ) is a FDA-approved anti-inflammatory drug used clinically in the treatment of rheumatoid arthritis and Crohn’s disease [16]. The mechanism underlying the biological effects of SFZ *in vivo* is complex and not completely understood. SFZ has immune-modulatory effects including inhibition of cyclooxygenase- and lipoxygenase-dependent pathways, enhancing anti-inflammatory adenosine release from sites of inflammation, and reducing leukocyte adhesion to endothelial cells [17,18]. This brief report presents the inhibitory activities of SFZ on SEB-activated human peripheral blood mononuclear cells (PBMC).

## 2. Results and Discussion

### 2.1. Effect of Sulfasalazine on Proinflammatory Mediators Release

**Figure 1:**
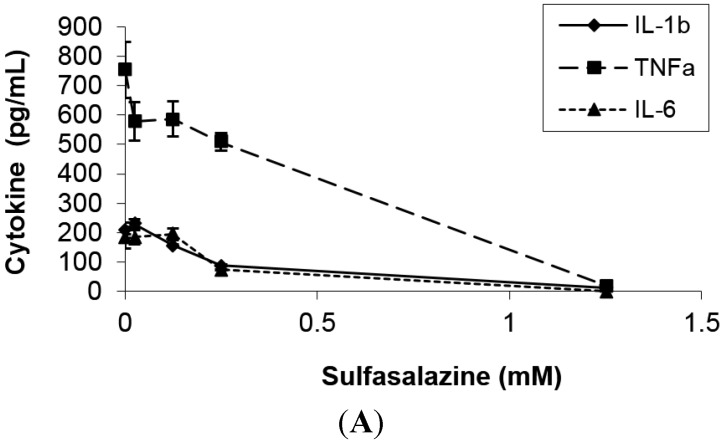

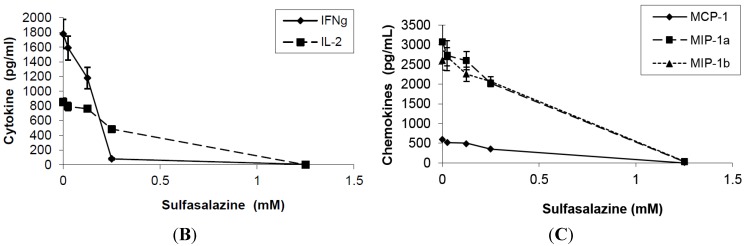
Dose-response inhibition of (**A**) interleukin 1β (IL-1β), tumor necrosis factor α (TNFα), and IL-6; (**B**) interferon γ (IFNγ) and IL-2; (**C**) monocyte chemotactic protein 1 (MCP-1), macrophage inflammatory protein (MIP)-1α, MIP-1β production by peripheral blood mononuclear cells (PBMC) stimulated with 200 ng/mL of staphylococcal enterotoxin B (SEB) in the presence of various concentrations of sulfasalazine (SFZ). Values represent the mean ± SD of duplicate samples and results represent three experiments. Results are statistically significant (*p* < 0.05) between SEB and SEB plus SFZ samples at concentrations of 0.25 and 1.25 mM.

The potency of SFZ in blocking cytokines and chemokines in SEB-stimulated human PBMC was investigated since proinflammatory mediators play key roles in superantigen-induced toxic shock. Figure 1 shows that SFZ attenuated the production of IL-1β, TNFα, IL-6, IL-2 and IFNγ in SEB-stimulated PBMC in a dose-dependent manner. The production of the chemokines, monocyte chemotactic protein 1 (MCP-1), macrophage inflammatory protein (MIP)-1α, and MIP-1β was also reduced. Reduction of these mediators were statistically significant (*p* < 0.05) between SEB and SEB plus SFZ samples at concentrations of 0.25 to 1.25 mM of SFZ. SFZ did not affect the viability of the cells over the concentration range used in these studies (0.025–1.25 mM), as demonstrated by trypan blue dye exclusion test. Lactate dehydrogenase assay also confirmed the lack of cytotoxic effects of SFZ in the concentrations used (data not shown).

### 2.2. Effect of Sulfasalazine on T-Cell Proliferation

**Figure 2:**
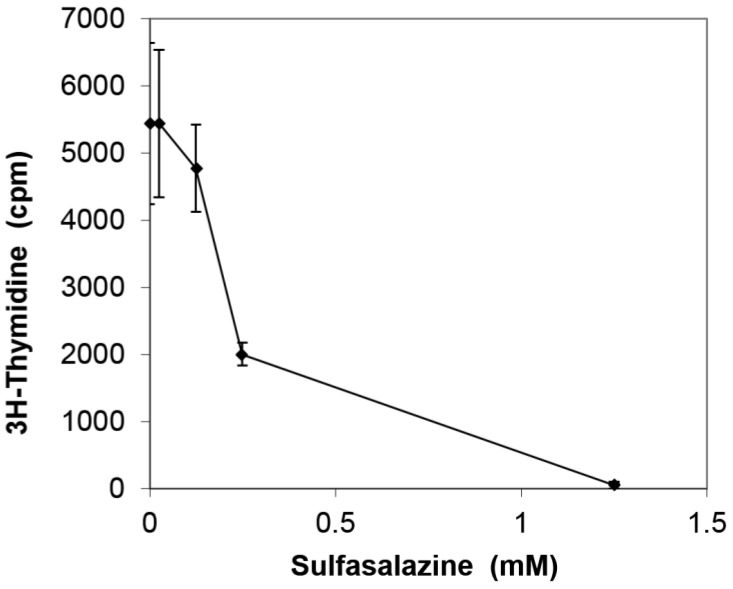
Inhibition of T-cell proliferation in PBMC stimulated with 200 ng/mL of SEB. Values represent the mean ± SD of triciplate samples and results represent three experiments. Results are statistically significant (*p* < 0.05) between SEB and SEB plus SFZ samples at concentrations of 0.25 and 1.25 mM.

Since SEB polyclonally activates T-cells, the effect of SFZ on SEB-stimulated T-cell proliferation was next examined. Figure 2 shows that SFZ effectively blocked T-cell proliferation, achieving 65% and 98% inhibition at 0.25 mM and 1.25 mM, respectively (*p* < 0.05).

### 2.3. Effect of Sulfasalazine on NFκB

**Figure 3:**
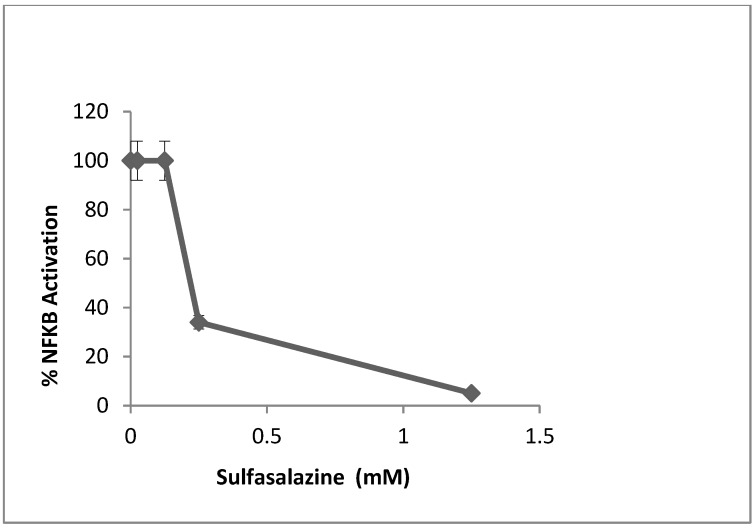
Inhibition of NF-κB activation in PBMC stimulated with 200 ng/mL of SEB. Values represent the mean ± SD of duplicate samples and results represent two experiments.

The transcription factor NF-κB is a key regulator of inflammation and acts downstream of many cell surface receptors including MHC class II molecules and cytokine receptors [19,20]. Cell extracts from SEB-stimulated PBMC in the presence of SFZ indicated that SFZ reduced NF-κB activation to 3% of control cultures of SEB-stimulated cells without drug treatment (*p* < 0.05, Figure 3).

The anti-inflammatory compound SFZ has been used clinically for decades to treat various inflammatory diseases such as rheumatoid arthritis and Crohn’s disease [16]. Its principal mode of action is inhibition of NF-κB, thereby down-regulating inflammation [21,22]. NF-κB is a key transcription factor involved in the regulation of a number of inflammatory cytokines, growth factors, and adhesion molecules [19]. A previous report indicated the activation of NF-κB in a human monocytic cell line treated with superantigens [23]. This study shows for the first time that SFZ reduces SEB-induced inflammatory mediators, T-cell proliferation and NF-κB.

## 3. Experimental Section

### 3.1. Materials

Purified SEB was obtained from Toxin Technology (Sarasota, FL, USA). The endotoxin content of these preparations was <1 ng of endotoxin/mg protein as determined by the Limulus amoebocyte lysate gelation test (BioWhittaker, Walkersville, MD, USA). Human (h) recombinant (r) TNFα, antibodies against hTNFα, peroxidase-conjugated anti-rabbit IgG, and peroxidase-conjugated anti-goat IgG were obtained from Boehringer‑Mannheim (Indianapolis, IN, USA). Human rIFNγ and rIL-6 were obtained from Collaborative Research (Boston, MA, USA). Antibodies against IFNγ, IL-2, and MCP-1 were obtained from BDPharMingen (San Diego, CA, USA). Recombinant IL-2, MCP-1, MIP-1α, MIP-1β; antibodies against IL-1β, IL-6, MIP-1α, and MIP-1β were purchased from R&D Systems (Minneapolis, MN, USA). SFZ and all other common reagents were purchased from Sigma (St. Louis, MO, USA).

### 3.2. Cell Culture

Human PBMC were isolated by Ficoll-Hypaque density gradient centrifugation of heparinized blood from normal human donors. PBMC (10^6^ cells/mL) were cultured at 37 °C in RPMI 1640 medium supplemented with 10% inactivated fetal bovine serum in 24-well plates as previously described [24]. Cells were stimulated with SEB (200 ng/mL) for 16 h. Varying concentrations (0.025, 0.125, 0.25, 1.25 mM) of SFZ were added simultaneously with SEB. Culture supernatants were collected and analyzed for IL-1β, TNFα, IL-6, IFNγ, IL-2, MCP-1, MIP-1α, and MIP-1β. Cell viability was determined by the trypan blue dye exclusion method. At the end of the experiments, cells were recovered and the number of trypan blue-positive cells was counted. Cells were 93%–98% viable in the presence or absence of SFZ with SEB using concentrations described above. Additionally, cell-free supernatants were also tested for the presence of lactate dehydrogenase, an enzyme that is released from dead cells.

T-cell proliferation was assayed with PBMC (10^6^ cells/well), which were plated in triplicate with SEB (200 ng/mL), with or without SFZ, for 48 h at 37 °C in 96-well microtiter plates. Cells were pulsed with 1 µCi/well of [^3^H]thymidine (New England Nuclear, Boston, MA, USA) during the last 5 h of culture as described previously [24]. Cells were harvested onto glass fiber filters, and incorporation of [^3^H]thymidine was measured by liquid scintillation.

### 3.3. Measurement of Cytokines and Chemokines

Cytokines and chemokines were measured by an enzyme-linked immunosorbent assay (ELISA) with cytokine- or chemokine-specific antibodies according to the manufacturer’s instructions, as previously described [24]. Human recombinant cytokines and chemokines (20–1000 pg/mL) were used as standards for calibration on each plate. The detection limit of each assay was 20 pg/mL. The cytokine and chemokine data were expressed as the mean concentration (pg/mL) ± SD of duplicate samples.

### 3.4. NF-κB Activation Assay

NF-κB activation was measured with a Trans-AM NF-κB kit (Active Motif, Carlsbad, CA, USA) according to the manufacturer’s instructions. Nuclear extracts (10 µg) containing NF-κB protein from PBMC with SEB in the absence or presence of SFZ were added to the wells, followed by the primary antibody against p65 subunit of NF-κB and the horseradish peroxidase-conjugated secondary antibody. Optical density was determined on an absorbance plate reader at 450 nm.

### 3.5. Data Analysis

Data were expressed as the mean ± SD and were analyzed for significant differences by the Student’s *t*-test with Stata (Stata Corp., College Station, TX, USA). Differences between SFZ-treated and untreated control groups were considered significant if *P* was < 0.05.

## 4. Conclusions

Development of medical countermeasures for preventing SEB-induced toxic shock is urgently needed to improve the high morbidity and mortality associated with complications from systemic shock resulting from bacterial superantigen exposure. Receptor blockade and signal transduction pathway inhibition represent different approaches to block superantigen-induced effects with various degrees of effectiveness [15]. Anti-inflammatory drugs are potentially useful as they target many downstream signaling pathways affecting multiple cytokines and chemokines. Repurposing FDA-approved drugs represent a fast approach for discovery of therapies against SEB and other biodefense related agents as safety concerns, tolerability, bioavailability and mechanism of action of these drugs are known. The new use of a FDA-approved anti-inflammatory compound, SFZ, against the biological effects of SEB is shown in this report. A logical extension of the inhibitory effects of SFZ on other staphylococcal superantigens may reveal broader applicability of its use and clinical potential. Further studies are underway to test the therapeutic efficacy of SFZ in various mouse models of superantigen-induced toxic shock.
